# Information Theory, Living Systems, and Communication Engineering

**DOI:** 10.3390/e26050430

**Published:** 2024-05-18

**Authors:** Dragana Bajić

**Affiliations:** Department of Communications and Signal Processing, Faculty of Technical Sciences, University of Novi Sad, Trg Dositeja Obradovica 6, 21000 Novi Sad, Serbia; dragana.bajic@gmail.com; Tel.: +381-11-24-36-441

**Keywords:** information theory, living systems, data transmission, synchronization, compression, error control, cryptography

## Abstract

Mainstream research on information theory within the field of living systems involves the application of analytical tools to understand a broad range of life processes. This paper is dedicated to an opposite problem: it explores the information theory and communication engineering methods that have counterparts in the data transmission process by way of DNA structures and neural fibers. Considering the requirements of modern multimedia, transmission methods chosen by nature may be different, suboptimal, or even far from optimal. However, nature is known for rational resource usage, so its methods have a significant advantage: they are proven to be sustainable. Perhaps understanding the engineering aspects of methods of nature can inspire a design of alternative green, stable, and low-cost transmission.

## 1. Introduction

We live in a digital era. Our access to multimedia for work and entertainment is virtually permanent. However, most users have never heard of Claude Shannon, although his works are the essence of worldwide communication connectivity.

The mathematical concepts established in 1948 in Shannon’s paper [1] announced a new discipline, with the title “information theory” (IT) premiered a year later [2]. Information theory owes its breakthrough to signal discretization based on another one of Shannon’s key contributions, the sampling theorem (1949) [3]. Admittedly, Shannon’s derivation on sampling was preceded by a conference paper by V. Kotelnikov [4]. The state of the art in 1933 could not envisage the implementation possibilities, so the work went unnoticed but not forgotten (having received the IEEE Alexander Graham Bell Award and the Eduard Rhein Foundation Award).

Nevertheless, Shannon’s works were the first to establish segments of information theory dealing with error control coding [5], cryptography [6], and compression [1], popularized by the Huffman code [7]. Digitalization soon became ubiquitous and has immense possibilities, from applications in multimedia that permeate speech, music, sounds, images, movies, and animated multidimensional reality to the sophisticated processing of medical signals and images that enable precise diagnostics.

The digital era implies that the previous one was analog. Indeed, telephony, television, radio, photographic material, gramophone records, video and audio magnetic tapes, and cassettes are some examples. Analog systems, however, have not been around forever. The first photographic negative dates from 1839, the first telephone conversation was in 1876, and the ECG was recorded in 1887 (the same year of inventing the “phonograph” (the predecessor of the gramophone)). The first X-ray image was taken at the end of 1895 and the first voiced radio message was recorded in 1897. In the 20th century, various analog audio, video, and even computing devices were invented, but their application was short-lived. In fact, the total lifespan of analog techniques was less than two centuries. The analog era was a significant period with pivotal technical achievements, but it was short-lived.

Preceding the analog era, discrete communication and recording systems were dominant techniques even then, although in a different form than nowadays. Transmission using Morse code was binary and line-of-sight transmission implemented binary signals generated by mirrors, fire, and smoke, while widespread storage systems (books) used discrete sets of symbols (letters). The pictographic records are also discrete, and only possessed a slightly higher number of symbols. Abacus, as a forerunner of computer systems, was discrete as well. Of all the other segments, cryptography existed even before Shannon incorporated classical ciphers into the framework of information theory. Compression based on dropping the vowels, practically based on (Shannon’s) entropy, was used at times when recording mediums was expensive (and it is still used in some languages). Morse code uses the same principles as Huffman’s algorithm, except that it was designed intuitively and does not follow the rules of a prefix code.

In an earlier period, eons before the formalization of information theory, while life on Earth was still emerging, nature has incorporated the concepts of information theory into its systems for transferring and storing discrete data through space and time.

This paper is dedicated to communication engineering methods that have analogies in the data transmission process by way of DNA structures and neural fibers. Considering the requirements of modern multimedia, transmission methods chosen by nature may be different, suboptimal, or even far from optimal. However, nature is known for rational resource usage, so its methods have a significant advantage: they are proven to be sustainable. Perhaps understanding the engineering aspects of such methods can inspire a design of alternative green, stable, and low-cost transmission.

Mainstream research on information theory within this field involves the application of genome analysis and, more generally, the analysis of a range of processes in living organisms. This is not a topic explored in this paper. This paper explores nature from the perspective of a communication engineer. However, for the sake of completeness, some very brief notions are included in Section 2.

Section 3 is devoted to nature’s interpretation of binary data based on the transmission of neurons in the body. Section 4 deals with the transmission and storage of DNA quaternary data and solutions that nature implemented for error control, cryptography, compression, and synchronization. Concluding remarks are given in Section 5.

## 2. Information Theory as a Tool

Few people know that Shannon’s doctoral dissertation was devoted to genetics [8]. It was a few years before the discovery of DNA when the classical Mendelian theory of inheritance still dominated. Shannon derived a general formula for the distribution of several related observed characteristic traits of individuals after a certain number of generations, assuming that the mating of individuals is random. The formula, although original and innovative at the time of receiving his Ph.D. (in 1940), went unnoticed, perhaps because the thesis was never published.In addition, Shannon changed his field of interest.

The first definition of information theory as a “general calculus for biology” was proposed in 1970 [9], but it is generally agreed upon that Lila Gatlin in 1971 was the first to thoroughly connect information theory to biological systems [10]. Her definition of life “… as an information processing system—a structural hierarchy of functioning units—that has acquired through evolution the ability to store and process the information necessary for its accurate reproduction” comprises complex IT-based mathematical analyses of DNA structures and diverse biological processes. One of the first analyses that implemented Shannon’s entropy was published in [11]. Since then, the mainstream of information theory in living systems has been to derive IT-based analytical tools for diverse genome and life system analyses. For example, one classical analysis study [12] explored how sensory stimuli are represented in the activity of neurons, combining techniques from physics, mathematics, and biology, with an emphasis on sensitivity to noise and errors, and concluded that it is not known if organisms perform the sort of reconstructions that yield favorable results in their analytical approach. The analysis carried out in [13] derives a theoretical framework for understanding the complex interplay between genetic information, regulatory proteins, and cellular processes, from the perspective of theoretical biophysics and computational neuroscience.

When considering the engineering aspects of works conducted by experts in information theory and error control coding, J. Hagenauer and his team stand out. They emphasized that their objective was to apply techniques from communications engineering to problems in the area of biology [14]. Their contributions were, among others, devoted to entropy analysis [15] and developing distance measures for the pairwise analysis of sequences of different lengths [16]. Several excellent review papers show the evolution of analytical methodologies conceived on the principles of information theory, with the first one being [17] (1996). Gene mapping was analyzed in [18,19], and, more recently, in [20].

However, recent review papers admit that IT might not always be an almighty analytical tool, as it requires sufficient sample sizes and significant computer resources, and both might be unavailable [21]. It is also noted that IT might not reveal which information is important for the organism, how biological systems use the information to carry out specific functions, and how a functional input–output relationship is established [22].

Nowadays, information theory is recognized as an integral part of understanding bio-systems and, in particular, DNA structures. It is the subject of collaborative research carried out by biologists, physiologists, mathematicians, physicists, and engineers, to mention only a few of the professions.

To bring back the paper focus, we refer to the inspiring works of T. Schneider [23,24,25], who, after speculating that “Claude Shannon probably never realized that his work on the mathematics of communications was about biology” [26], contributed to our hypothesis that nature might guide us towards an engineering solution: “All the sophisticated mechanisms of biology must be based on codes—we ‘just’ have to learn what those codes are so that we can apply them to our own technologies” [27].

## 3. Small-Scale Transmission in the Body: Binary Data

**Figure 1:**
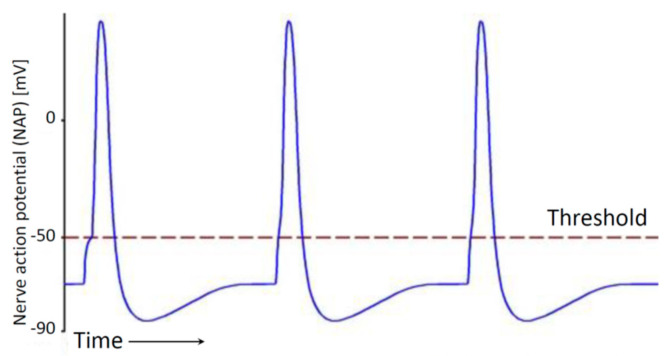
Pulses of nerve action potential (NAP)—information is transferred using digital frequency modulation.

A mechanistic approach to the functionality of data transmission within the human body reduces it to a wired electrical transmission along the neural lines. This over-simplified statement evoked a lot of protests, as other systems, such as renal and endocrine systems, are also responsible for data transmission. However, the dominant transmission path is neurons, and the carriers of information are impulses of nerve action potential (NAP) (Figure 1).

It was generally assumed that the NAP amplitude and shape are a constant for a particular neuron. The information is proportional to the number of impulses per unit of time, that is, to frequency. A stronger signal indicates more pulses and a weaker signal indicates fewer pulses, which means that frequency modulation is responsible for the transmission of information via nerve fibers. Signaling is binary, with NAP impulses being either “on” (if a threshold is exceeded) or “off” [28]. In [29], the process of converting information in the retina is modelled as an analog-to-digital convertor. Synapses were considered to be mere connectors, where transmission slows down as it is performed using chemical diffusion (similar to electrical connectors that attenuate the pulse energy).

More recent findings, however, show that the pulse shape, initially believed to be constant for a particular neuron, can be changed in multiple ways [30]. Data transfer is shown to have an analog component as, in addition to digital action potentials, graded subthreshold synaptic potentials propagate as well [30,31,32,33]. Hybrid analog–digital transmission shows that the amount of transferred information is larger than initially supposed. In data transmission systems, a similar simple but purely digital approach is implemented in the form of so-called “piggybacking”, where low-rate binary data are embedded into high-rate binary data [34]. This incites an idea for further improving communication systems: to enable continuous (analog) changes in the amplitude (or some other parameters of digital signals) and provide hybrid transmission that might increase data flow or protect data in a steganography manner.

Digital frequency modulation is not the only analogy. Nature has improved transmission by applying destructive (lossy) compression. The relationship between excitation and pulse frequency is not linear and can be approximated by non-linear functions. The most frequent approximation is logarithmic, as the relationship between excitation *S* and pulse frequency *f* is expressed by the Weber–Fechner law, according to which *f* ≈ a·log(*S*) + b, where a and b are empirical constants [28]. Logarithmic transformation reduces the amplitude range so the transmission can be performed in real time. For example, the interval between neural impulses is physiologically limited (about 5 ms in humans), and the strongest excitation to which such an interval corresponds is considered to be typically 10^6^ times greater than the weakest. If the connection was linear, the interval corresponding to the smallest excitation would be 10^6^ times longer than 5 ms, so the time needed to feel a light excitation, for example, a feather falling on the hand, would be more than an hour. The price to be paid for more efficient transmission is low resolution. To distinguish between two different stimuli, a greater change in their intensity is required.

Destructive nature’s ability to compress our senses has a great impact on how we save resources for real communication data transmission and storage. It provides the possibility of eliminating information content that our senses are unable to perceive. The first benefit relates to digital telephone signals, where technical interfaces were required to transfer the data with a mere rate of 64 kb/s (8 bits per sample), although satisfactory quality required at least 12 bits per sample, equivalent to 96 kb/s. This goal was achieved via the nonlinear attenuation of large speech amplitudes that correspond to vowels which are insignificant considering the information content [35].

Another benefit of nature’s ability to compress our senses regards the savings in storage resources. Numerous standardized destructive compression algorithms reduce the size of bulky data, such as images, video, and audio signals. These algorithms designed by humans are tuned to the compression algorithm designed by nature so that the eye or ear of the user cannot perceive any distortion.

## 4. Large-Scale Transmission across Space and Time: Quaternary Data

The DNA molecule is a memory block that stores genetic records. It is a very long molecule composed of millions of constitutive elements—nucleotides. There are only four different nucleotides, labeled A, G, T(U), and C, drawn from a four-symbol alphabet [36]. Information is stored (and transmitted) as a sequence of nucleotides, and complexity is achieved by their large number. DNA transmits information across space, via the movement of the host, and across time, via inheritance. DNA records are permanent—the DNA record of a Siberian mammoth has been successfully decoded, although it lived around 1,500,000 years ago [37].

### 4.1. DNA-Based Signals

A DNA molecule consists of two chains of nucleotides (two strands). Parallel nucleotides are connected according to the template A-T and G-C so that the strands are complementary. A human body contains 10^9^ nucleotides (discrete carriers of information), and the total length of the DNA molecule is equal to 400 distances from the earth to the sun [36].

There is ambiguity surrounding whether the genetic record initially only contained binary nucleotides [38] and whether they were slowly transferred towards quaternary symbols. The basis of this hypothesis is that the binary alphabet is the simplest, and life began with the simplest organisms. Simplistic reasoning without evidence caused negative reactions, but no conclusion was drawn because neither side provided evidence.

The engineering logic would be that the number of binary nucleotides would be the square of the number of quaternary nucleotides (10^18^ instead of 10^9^ nucleotides in humans). The DNA strands would be too long for comfortable handling; the replication process would require more organic material and the translation process would require a lengthy search along the strands, so more energy would be consumed if the data were binary.

Such reasoning implies that an increased number of nucleotides would have shorter chains and achieve greater energy and material savings. However, nature stopped at four nucleotides. A parallel may likely be found in communications design. A change in the number of signaling levels from four to eight could be performed only if a considerable hardware change was made. This occurred in dial-up modems. To accomplish the eight-level modulation with the desired rate, the classical step-by-step signal generation had to be replaced by a signal processor that directly synthesized the waveforms [39]. It may be speculated that an increase in the number of different nucleotides makes the DNA “hardware” unstable.

**Figure 2:**
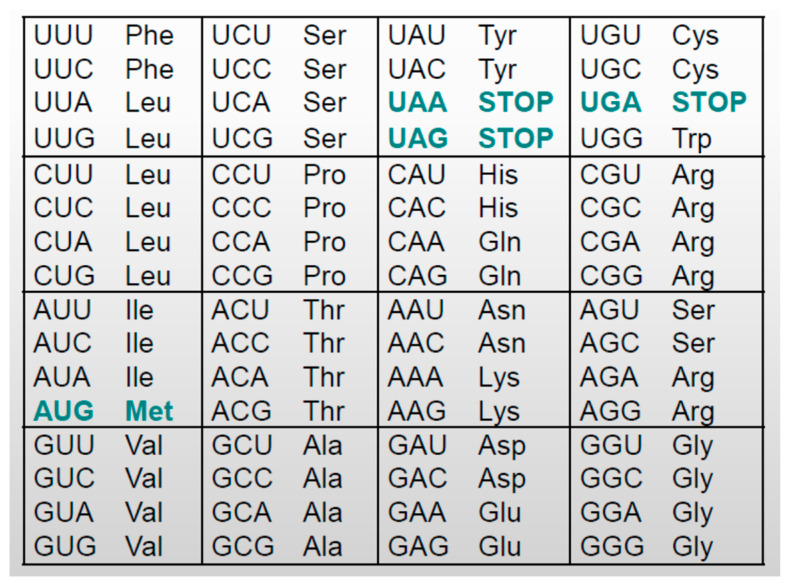
Codons—triplets of nucleotides. Here, 1, 2, 3, 4, or 6 codons can be mapped into the same amino acid. The codons UAA, UAG and UGA are not amino acids, they mark the end of transcription process, while amino acid Met sometimes mark the beginning.

Nucleotides are organized in triplets called codons. There are 4^3^ = 64 possible triplets, mapped into amino acids and into so-called “stop” or “nonsense” codons, according to Figure 2. A sequence of amino acids makes up a protein, which is a key factor in all processes in living beings. Information on each protein is written in segments of the DNA molecule. Those segments represent the gene for the given protein [36].

This hierarchical system (in the order of nucleotides, amino acids, genes, and proteins) resembles the hierarchy of digital transport systems, where the basic information carriers are gradually multiplexed into higher-level entities via low-level containers, from basic input signals with lower data rates to high-level containers with higher data rates, ending with the entire frame.

### 4.2. Error Control

The transfer of data to a new cell occurs with each cell division in a procedure called DNA replication. As with any type of data transfer, it is prone to errors, for which the only form of protection is inserting the information redundancy. The nature provided this redundancy via a simple repetition, whereby DNA comprises two complementary strands, so the data are “doubled”.

During the replication stage, the DNA strands are separated, and the DNA polymerase enzyme begins to act on each nucleotide, generating its complement. Several types of errors can occur. The first type is a substitution error when the wrong nucleotide is generated, either if an added nucleotide is in a rare form (tautomer) or if the nucleotide is slightly shifted (wobble). The second type of error is strand slippage, when either existing or newly synthesized strands make a loop, causing the deletion of an existing nucleotide or the insertion of a non-existing nucleotide [40]. Both of these errors have their counterparts in communication data transmission. The first one is simply an error. The second one is known as a “cycle-slip”; if the clock rate at the receiver is too high or too low, additional symbols would be inserted or some of the received symbols would be “missed”.

In the process of DNA replication, DNA polymerase makes a mistake once in every 100,000 nucleotides. Knowing that one cell can contain up to 6 billion pairs of nucleotides, up to 120,000 errors are generated during just one cell division [40].

Errors are corrected by the DNA polymerase itself in a procedure called “proofreading”, which recognizes irregular strand bonding and removes errors based on informational redundancy in chemical compounds. Thus, about 99% of errors are removed. This is insufficient. Residual errors are subject to the second level of error correction, “mismatch repair”, where several different enzymes look at the entire DNA structure and replace the wrong nucleotide with the correct one. The residual error after the procedure is about 10^−9^ [40].

In comparison, an error probability of 10^−5^, according to standards [41], is considered a link failure in digital transport systems with a synchronous digital hierarchy (SDH) that globally carry data via (mostly) optical links. However, there is a difference between the significance of the residual errors in communication theory and the DNA data transfer. Communication engineers would be happy to eliminate all the errors, while nature tends to preserve some of them. Most DNA errors lead to mutations, which usually have a negative connotation but also provide genetic variability that is a prerequisite to evolution.

Another form of error control coding occurs at a higher hierarchical level. Nature performs the unequal error control of amino acids, coding all but two of them by two, three, four, or six different codons. For example, the amino acid alanine is coded by codons GCU, GCC, GCA, and GCG. A tautomeric error translates A to C, so GCA becomes GCC. However, both GCA and GCC are codewords for alanine, so even if the error at the first level at DNA replication is not corrected, the gene for this protein remains the same.

### 4.3. Cryptography

One-way functions, in the context of communications and cryptography, are mathematical functions that are easy to compute in one direction but difficult to reverse. In [42] (p. 193), it is said that the existence of one-way functions is equivalent to the existence of all (non-trivial) private-key cryptography, and this constitutes one of the major contributions of modern cryptography. As a simplest example, it is easy to compute a product of two integers, but, given a product, its factorization in the integer ring is not easy. They are also related to the ciphers with an asymmetric (public) key [43], which are considered one of the most significant breakthroughs in the history of world cryptography [34].

The information on proteins is protected on the principle of one-way function. Mapping the codon to an amino acid is easy, but reverse mapping is not. From the previous section, it is obvious that the same amino acid is mapped from different codons (Figure 2). A simple example reveals that if there were 100 amino acids per protein, formed by only two different codons each, there would be 2^100^ ≈ 10^30^ different possibilities to generate the same protein. It is not clear, however, if such protection is unintentional but accidentally achieved as a consequence of unequal error protection or if nature has designed it with a true security purpose and, if so, against whom.

The reverse problem is the human-designed protection of DNA data. The need exists because silicon-based storage media are expected to outstrip available resources. The solution already taken from nature is DNA storage that is rapidly developing [44]. Stored data require protection, so efforts are aimed at designing a cryptography system targeted for data with DNA molecular structures [45,46], implementing a one-time pad system that is more feasible in DNA than in classical systems [47], and creating a system based on the characteristics of DNA molecules [48,49]. Such systems are expected to potentially complement traditional and quantum cryptography [50].

### 4.4. Compression

Although the number of nucleotides that define the genome is impressive and provides a considerable data storage, nature has made savings possible. Contrary to the destructive compression of small-scale transmission, in the case of DNA, the compression must be non-destructive (lossless). Such a compression reduces the necessary resources while retaining the same amount of information. It is achieved by “folding” individual genes, using the same nucleotides of the DNA strand to code for different genes. This is especially characteristic of viruses because their genomes are small, but all the same, they must store a lot of data [51,52]. This also occurs in the human genome where 774 overlapping gene pairs have been found [33].

**Figure 3:**
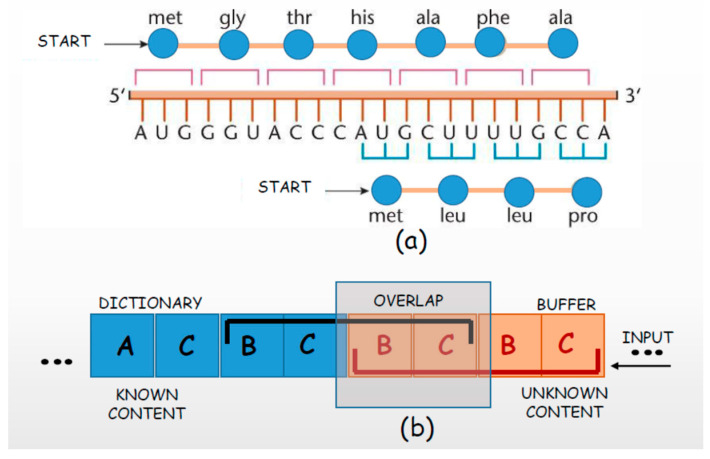
Non-destructive compression. (**a**) Two overlapping genes with differently synchronized codons; (**b**) overlapping the contents of dictionary and input buffer in the Ziv–Lempel 77 procedure.

Figure 3a shows the start of two different genes in the same DNA strand [36]. Both genes use the same nucleotides, but the corresponding codons are organized differently. A codon consists of three nucleotides, so there are three possible starting positions. In the example shown in Figure 3a, the beginnings of the codons of the two genes are not synchronized, and the first nucleotide of the codon in the “lower” gene is shifted by one position relative to the codons in the “upper” gene.

The same overlap model is applied in the LZ77 (Ziv–Lempel 77) dictionary technique for non-destructive compression [53]. An example is shown in Figure 3b. Information already encoded is stored in the dictionary, while information awaiting compression is stored in the input buffer. Encoding involves finding the input string in the dictionary and registering its position and length. In this example, the specific string BCBC is not in the dictionary. But, its bifix structure [54,55] enables compression since the suffix of the string that is not in the dictionary overlaps the contents in the input buffer. This overlapping enables a higher degree of compression (Figure 3b).

However, regarding DNA compression, another problem arose. Advances in DNA analysis and sequencing caused an exponential increase in genomic data, as shown in [53]. Its storage requires lossless compression. At first, the classical Ziv–Lempel (LZ) compression algorithm seemed to be sufficient. Then, an algorithm based on statistical evolutionary models and prediction techniques from lossless binary image compression was introduced. It outperformed the LZ algorithm by a factor of 1.6 because it was designed and tuned exclusively for genome data [56].

### 4.5. Synchronization

**Figure 4:**
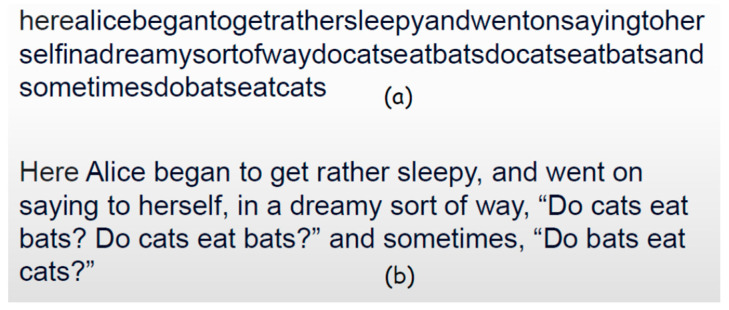
(**a**) Non-synchronized stream of digital symbols; (**b**) the same text with included blanks, capital letters, and punctuation marks to differentiate (synchronize) words and sentences by denoting their beginnings.

Synchronization is a key function of digital systems. In a stream of digital data, it is almost impossible to find the beginning of the message, unless it is marked by a synchronization sequence (sync marker). An illustrative example is shown in Figure 4. Ordinary text is full of sync markers (Figure 4b): “blank” marks the beginning and end of the word, while capital letters and punctuation signs mark the beginning and end of the sentences. Without those sync markers, ordinary text (Figure 4a) is quite unintelligible.

In communication transmission systems, data are binary and framed, i.e., the beginning of the stream of data is, without exception, marked by a sync sequence. Data can be de-multiplexed and read if a sync sequence is found. The end of the data stream need not be marked as it depends on the protocol.

**Figure 5:**
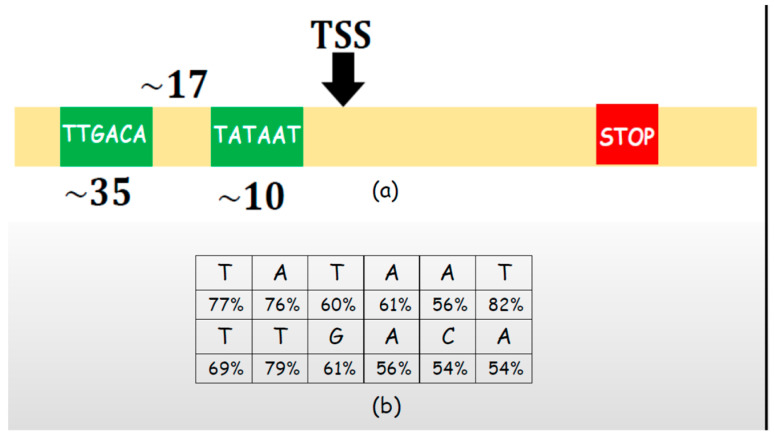
(**a**) Transcription start site (TSS) and markers TATAAT and TTGACA at the offset of approximately 10 and 35 positions from TSS; (**b**) probability that the nucleotide is at its expected position.

The stream of DNA data contains the information on the proteins. To replicate the protein, it is necessary to find the corresponding gene where the data are stored. It is performed in the “transcription” process and involves finding the exact position of the gene within the DNA strand. This position is called the “transcription start site” (TSS) (Figure 5a). The TSS position of the particular gene is preceded by the “promoter region” that contains the information on the TSS position.

An enzyme RNA polymerase “slides” along the DNA chain until it reaches the start of the protein gene. This procedure is similar to the serial acquisition of sync markers in synchronous transport systems. Synchronization has been mostly analyzed in relation to *Escherichia coli* bacteria, which is the simplest case [57,58,59,60].

However, the promoter region is not too helpful. Its information does not provide unequivocal instructions on how to find TSS. There are two sync markers in the promoter region, each comprising six nucleotides (Figure 5a). Contrary to communication systems where the structure and position of markers are always the same, the position and structure of sync markers in DNA are not firmly defined. Probabilities of sync nucleotides from Figure 5a are shown in Figure 5b. The decision that the markers are found is made by consensus (for this reason, the sync markers are frequently called “consensus” sequences). Finding the markers does not mean that the position of TSS is known. The mutual distance of the two markers is 16, 17, or 18 with a probability of 92%, and the remaining 8% corresponds to distances of 12, 13, 19, 20, etc.

Even worse, TSS is located with a probability of 75% at a distance of 7 ± 1 from the sequence marked with −10. The experiments so far have not found a solution to how the enzyme can find TSS, given that, in the remaining 25% of cases, distances show larger deviations, and, to emphasize again, E. Coli is the simplest sample.

From the previous example, it is obvious that the synchronization process for transcription is not performed analogously to synchronization in digital communications. The synchronization rules are considerably more relaxed when compared to the rigid ITU-T (International Telecommunication Union—Telecommunication Standardization Sector) recommendations. However, these relaxed rules have successfully transcribed proteins from their genes for millions of years, and it would be a challenge to answer “why” this happens.

However, a segment where DNA synchronization outperforms human synchronization systems is the sync marker that marks the end of the data stream (i.e., the end of the frame).

Data frames in computer communication most often comprise a “STOP” byte to mark the end of the frame. Framing and synchronization in this context are specified by the Open Systems Interconnection (OSI) model, a reference model of the International Organization for Standardization. The aforementioned functions belong to the second of seven functional layers, known as the data link layer.

The stream of information data is without constraints, and the information byte can be the same as the STOP byte and cause an error in the end of the frame. So, another redundant byte is inserted to show that the information byte that follows is just incidentally equal to the STOP byte. This redundant byte should be ignored at the receiver, but if it is simulated by data bytes, it cannot be ignored, i.e., it should be marked as information by an additional redundant byte. The complete procedure is known as “byte stuffing” [34].

Nature has avoided byte stuffing. Figure 2 shows STOP codons; any of them unambiguously mark the end of the transcription. These codons cannot become amino acids and cannot falsely mark the end of the transcription.

Sometimes, the translation continues past the stop codon, a phenomenon called “stop-codon read-through”. This is not a rare occurrence, and it has prompted the adaptive hypothesis that it is an important regulated mechanism. More recent research has doubted that such events have biological functions and suggested that most of them are non-adaptive cellular errors [61]. If this is the case, it would correspond to the communication event when channel errors corrupt the STOP byte and the receiver continues to receive non-informative data.

## 5. Discussion

This paper outlined some of the transmission and storage problems in living systems. Some of the solutions are almost equivalent. Some are simple, in particular error control, which is a simple repetition coding the same information by several codewords. Some did not fall into the trap that (computer) engineers tripped over. Some are different. But, nature is diverse, and so the more observant we are and the more we consider its engineering, the more we shall know about how to think differently and apply them. Nature has worked for years, and its engineering solutions should be seriously listened to and considered.

## Data Availability

Not applicable.
